# Suicidality in adults with obstructive sleep apnea: A systematic review and Meta-Analysis

**DOI:** 10.1007/s11325-025-03462-5

**Published:** 2025-09-29

**Authors:** Lauren R. McCray, Anna J. Kulangara, Shaun A. Nguyen, Ted A. Meyer, Robert F. Labadie, Mohamed A. Abdelwahab

**Affiliations:** 1 Department of Otolaryngology-Head and Neck Surgery, Medical University of South Carolina, 135 Rutledge Avenue, MSC 550, Charleston, SC 29425 USA; 2 Baylor College of Medicine, Houston, TX USA

**Keywords:** Obstructive sleep apnea, Quality of life, Meta-analysis, Systematic review

## Abstract

**Purpose:**

To assess relations between obstructive sleep apnea (OSA) and suicidality, measured by suicidal ideation, suicide attempts, and death by suicide.

**Methods:**

CINAHL, Cochrane Library, PubMed, PsycINFO and SCOPUS were searched from inception through June 24, 2024. Observational studies related to suicidality in adult OSA patients were included. Case reports and studies on central sleep apnea were excluded. Data were extracted and reviewed by two authors, with disagreements resolved by a third. Risk of bias was assessed with the Risk Of Bias In Non-randomized Studies - of Exposure for cohort studies and the Joanna Briggs Institute checklist for case-control and cross-sectional studies. Random effects meta-analyses (single means, proportions, and relative risks (RR)) were performed.

**Results:**

Fifteen studies (*n* = 1,438,523) were included: eight assessed suicidal ideation, two assessed suicide attempts, and six assessed death by suicide. The OSA group experienced a higher prevalence of suicidal ideation (12.4% [95% CI: 8.9%-16.3%] vs. 3.7% [95% CI: 1.9%-6.2%]), suicide attempts (1.5% [95% CI: 0.9%-2.2%] vs. 0.9% [95% CI: 0.1%-2.2%]), and death by suicide (0.3% [95% CI: 0.2%-0.4%] vs. 0.2% [95% CI: 0.1%-0.3%]) than controls (*p* < 0.001). The OSA group had a higher risk of suicidal ideation (RR = 1.8, 95% CI: 1.5–2.1) and death by suicide (RR = 1.9, 95% CI: 1.1–3.4) than the control group (*p* < 0.001).

**Conclusion:**

Suicidal ideation affects nearly 12% of patients with OSA, with close to 2% attempting suicide. Otolaryngologists should consider the risk of suicidality in these patients.

**Supplementary Information:**

The online version contains supplementary material available at 10.1007/s11325-025-03462-5.

## Introduction

Obstructive sleep apnea (OSA) affects approximately 9–38% of the general adult population, with variations depending on age, sex, and body habitus [1]. The disorder is associated with serious adverse health outcomes, including cardiovascular diseases, metabolic syndrome, and neurocognitive impairments, resulting in increased healthcare utilization [2, 3]. Recent research has highlighted a concerning association between OSA and mental health disorders, particularly depression and anxiety [4]. However, the link between OSA and suicidality, including both suicidal ideation and suicide attempts, has received less attention.

The mechanisms underlying the association between OSA and suicidality may involve neurobiological, psychological, and social factors. Suicidality in individuals with OSA may be influenced by the cumulative burden of sleep fragmentation, chronic fatigue, and hypoxemia, which can exacerbate psychological distress and reduce coping mechanisms [5]. The subsequent hypoxemia and sleep fragmentation can lead to neuroinflammation and oxidative stress, contributing to the pathophysiology of depression and suicidal behavior [6]. Additionally, chronic sleep deprivation associated with OSA may impair emotional regulation and decision-making, further contributing to suicidal ideation [7].

The aim of this systematic review and meta-analysis is to investigate whether adults with OSA have an elevated risk of suicidality. We also sought to evaluate how the prevalence of suicidality differed based on treatment modalities and gender. By synthesizing the available data, we aim to provide a better understanding of the risk of suicide in the OSA population to inform clinical practice and guide future research efforts.

## Methods

### Data collection and selection

This study adhered to the Preferred Reporting Items for Systematic Reviews and Meta-Analyses (‘PRISMA’) guidelines [8]. Our aim was to compare the prevalence of suicidality among adults with OSA to adults without OSA, as measured by suicidal ideation, suicide attempts, and death by suicide. To identify relevant studies, authors LRM and AJK searched PubMed (National Library of Medicine – National Institutes of Health), Scopus (Elsevier), CINAHL Complete (EBSCOhost), PsycINFO (American Psychological Association), and Cochrane Library (Wiley) databases from inception through 24 June 2024. Tailored search strategies were employed for each database, combining medical subject headings with the following keywords: obstructive sleep apnea, suicide, anxiety, depression, self-harm, and mental illness. Details of the search strategy can be found in the supplemental materials. Additionally, reference lists of pertinent articles were reviewed. All retrieved citations were imported into Covidence (Veritas Health Innovation Ltd., Melbourne, Australia) and independently screened for eligibility by LRM and AJK.

## Selection criteria

Studies were eligible if they assessed suicidality and mental health outcomes in OSA patients at least 18 years old. Exclusion criteria included non-English publications, research involving non-human subjects, and conference abstracts. Both cross-sectional and cohort study designs were considered, whereas case studies and case reports were not included.

## Data extraction

Information on study characteristics, including author and publication year, as well as participant demographics such as age and sex, was collected. The prevalence of mental health outcomes, including anxiety, depression, suicidal ideation, suicide attempts, and death by suicide, was also recorded for analysis. The level of evidence for each included study was assessed according to the Oxford Centre for Evidence-Based Medicine criteria [9].

## Quality assessment

The risk of bias for cohort studies was evaluated using the Risk Of Bias In Non-randomized Studies – of Exposure (ROBINS-E) tool [10], while the Joanna Briggs Institute (JBI) critical appraisal checklists were applied to cross-sectional and case-control studies [11, 12]. For ROBINS-E, each domain of bias was rated as low, unclear, or high. The JBI checklists include eight items for cross-sectional studies and ten for case-control studies. Each item was scored as “1” for “yes” and “0” for “no,” “not applicable,” or “unclear.” We categorized studies as low risk of bias if they achieved a score of four or higher for cross-sectional studies, or five or higher for case-control studies. One author (LRM) conducted the initial risk-of-bias assessment for all studies included in the meta-analysis, with a second author (AJK) verifying the evaluations. Any disagreements were resolved with input from a third author (SAN). Assessed risk-of-bias domains included inclusion criteria, selection bias, exposure measurement, confounding, outcome measure validity and reliability, appropriateness of statistical analyses, and other relevant factors.

### Statistical analysis

Meta-analysis of continuous measures (age) was performed with Comprehensive Meta-Analysis version 4 (Biostat Inc, Englewood, NJ, USA). Meta-analysis of risk ratio (OSA group vs. control group) for suicidal ideation, suicidal attempts, and death by suicide was performed with Cochrane Review Manager (RevMan) version 5.4 (The Cochrane Collaboration 2020, United Kingdom). Meta-analysis of proportions (patient characteristics, prevalence, etc.) was performed using MedCalc 22.017 (MedCalc Software, Ostend, Belgium). Each measure (mean, proportion [%], risk ratio [RR] and 95% confidence interval [CI]) was weighted according to the number of patients affected. Heterogeneity among studies was assessed using I^2^ statistics with fixed effects (I^2^ < 50%) and random effects (I^2^ > 50%). In addition, a comparison of proportions, expressed as difference (Δ) and 95% CI was done to compare outcomes between two groups. Potential publication bias was evaluated by visual inspection of the funnel plot and Egger’s regression test, which statistically examines the asymmetry of the funnel plot. A p-value of less than 0.05 was considered a statistically significant difference for all statistical tests [13, 14].

## Results

### Study characteristics

**Fig. 1:**
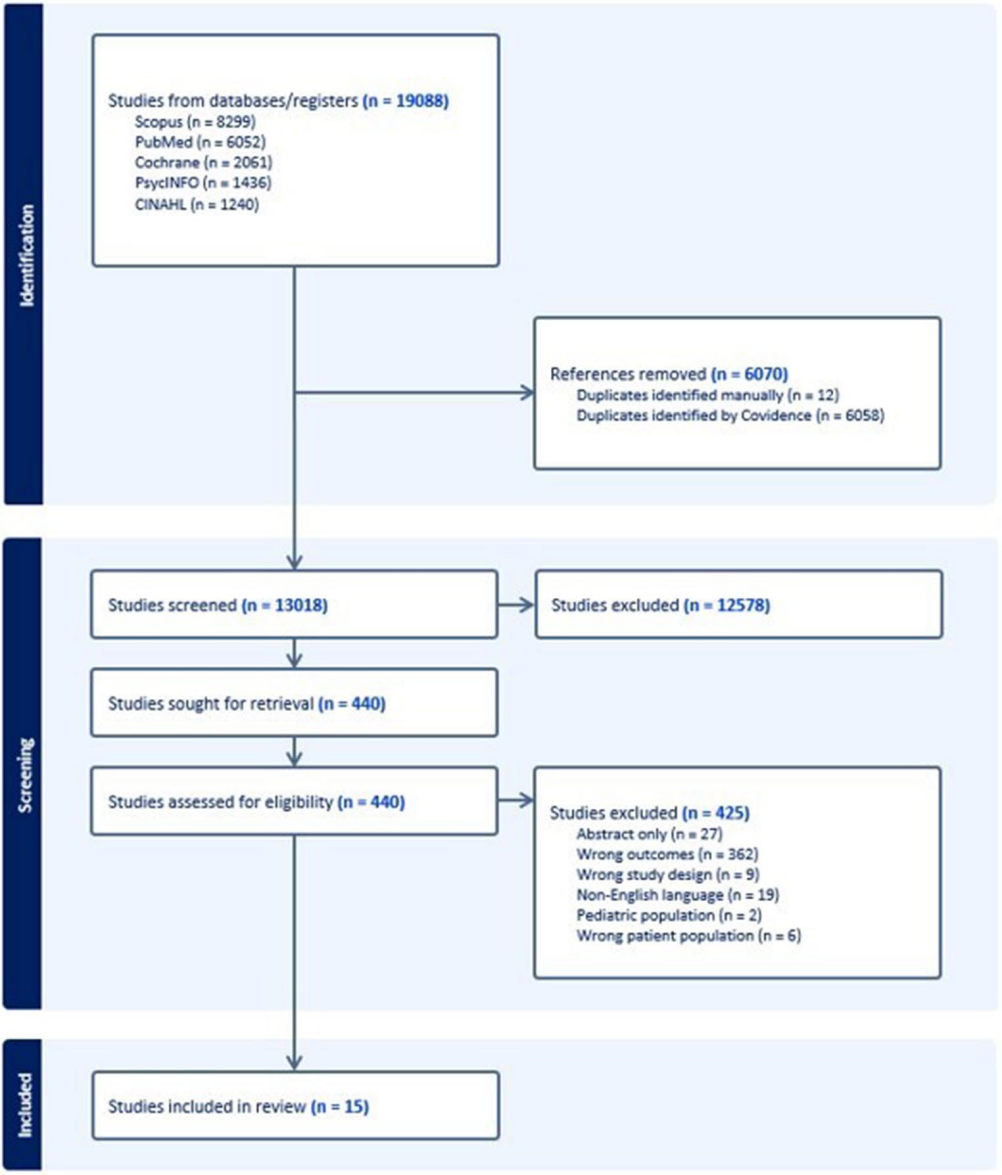
Flowchart depicting study attrition through screening and eligibility assessments

After eliminating duplicates, 13,018 titles and abstracts underwent screening for eligibility. Of those studies, 424 underwent full-text review, and fifteen were ultimately included for analysis (Fig. 1) [15–29].

The included studies, published between 1995 and 2023, comprised a total of 1,438,523 participants. Of these, 215,500 had OSA confirmed by medical records or polysomnography (PSG). An additional 6,653 participants self-reported an OSA diagnosis from a medical professional within the past 12 months, 409 reported a lifetime diagnosis of OSA, and 1,397 reported symptoms consistent with sleep-disordered breathing. The remaining participants served as controls without reported or diagnosed OSA.

The OSA population was 73.9% male (95% CI: 71.1% − 76.7%) with an average age of 52.1 years (95% CI: 45.8–58.4; range: 20–71 years), an average AHI of 41.3 (95% CI: 31.7–51.0), and an average BMI of 34.1 (95% CI: 31.5–36.6). The control population was 63.5% male (95% CI: 52.1% − 74.2%) with an average age of 55.2 years (95% CI: 37.6–72.8 years). The studies represented a diverse range of countries, with four from the United States, two from Denmark, two from Taiwan, and one each from South Korea, Sweden, Slovakia, Africa, Israel, Italy, and France. Table 1 summarizes key characteristics of the included studies.

**Table 1:** Characteristics of included studies and patient cohorts

Study (year)	Country	OLE	OSA Patients (*n*)	Males (*n*)	Patient age in years (Mean (SD))	Suicidality Outcome	Method of Assessing Suicidality
Bishop et al. (2018) [15]	United States	4	1155	659	-	Suicidal ideation; suicide attempt	Self-reported over past 12 months
Cheng et al. (2021) [16]	Taiwan	3	6915	4640	-	Death by suicide	ICD-9 codes
Choi et al. (2015) [17]	South Korea	4	117	102	49.4 (10.3)	Suicidal ideation	Item 9 on BDI-II
Chu et al. (2023) [18]	Taiwan	3	7095	5580	46.2 (12.8)	Suicide attempt	ICD-9 codes
Gharsalli et al. (2022) [19]	Africa	4	80	28	54.8 (13.1)	Suicidal ideation	Self-reported; unspecified time period
Hashmi et al. (2006) [20]	United States	4	98	98	53.2 (10.1)	Suicidal ideation	Item 9 on BDI-II
Hoier et al. (2022) [21]	Denmark	3	70,889	52,885	-	Death by suicide	ICD-8 and ICD-10 codes
Kaufmann et al. (2017) [22]	United States	4	5498	3299	-	Suicidal ideation	Self-reported over past 12 months
Lavie et al. (1995) [23]	Israel	3	1620	1456	48.1 (11.0)	Death by suicide	Death certificate
Pierobon et al. (2008) [24]	Italy	4	157	106	47.8 (11.9)	Suicidal ideation	CBA 2.0
Rod et al. (2017) [25]	Sweden	3	74,543	55,112	-	Death by suicide	ICD-10 codes
Timkova et al. (2020) [26]	Slovakia	4	149	101	49.0 (9.6)	Suicidal ideation	GHQ-28
Udholm et al. (2022) [27]	Denmark	3	48,168	37,427	64.2 (13)	Death by suicide	ICD-10 codes
Veale et al. (2000) [28]	France	4	5669	4874	55.9 (11.0)	Death by suicide	Medical records
Wheaton et al. (2012) [29]	United States	4	1806	1081	-	Suicidal ideation	PHQ-9

*BDI-II *Beck Depression Inventory–Second Edition, *CBA 2.0 *Cognitive Behavioral Assessment 2.0, *GHQ-28 *General Health Questionnaire–28 item version, *ICD *International Classification of Diseases, *OLE *Oxford Level of Evidence, *OSA *obstructive sleep apnea, *PHQ-9 *Patient Health Questionnaire–9

## Quality assessment

**Fig. 2:**
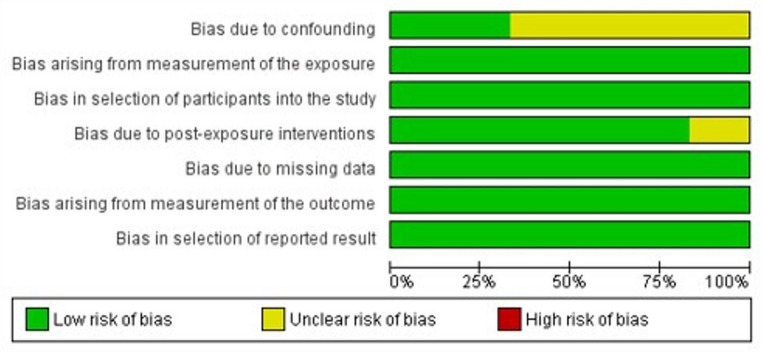
Risk of bias assessment. Proportions of studies assigned to each risk category for individual bias items are presented, as assessed using the ROBINS-E Tool

**Fig. 3:**
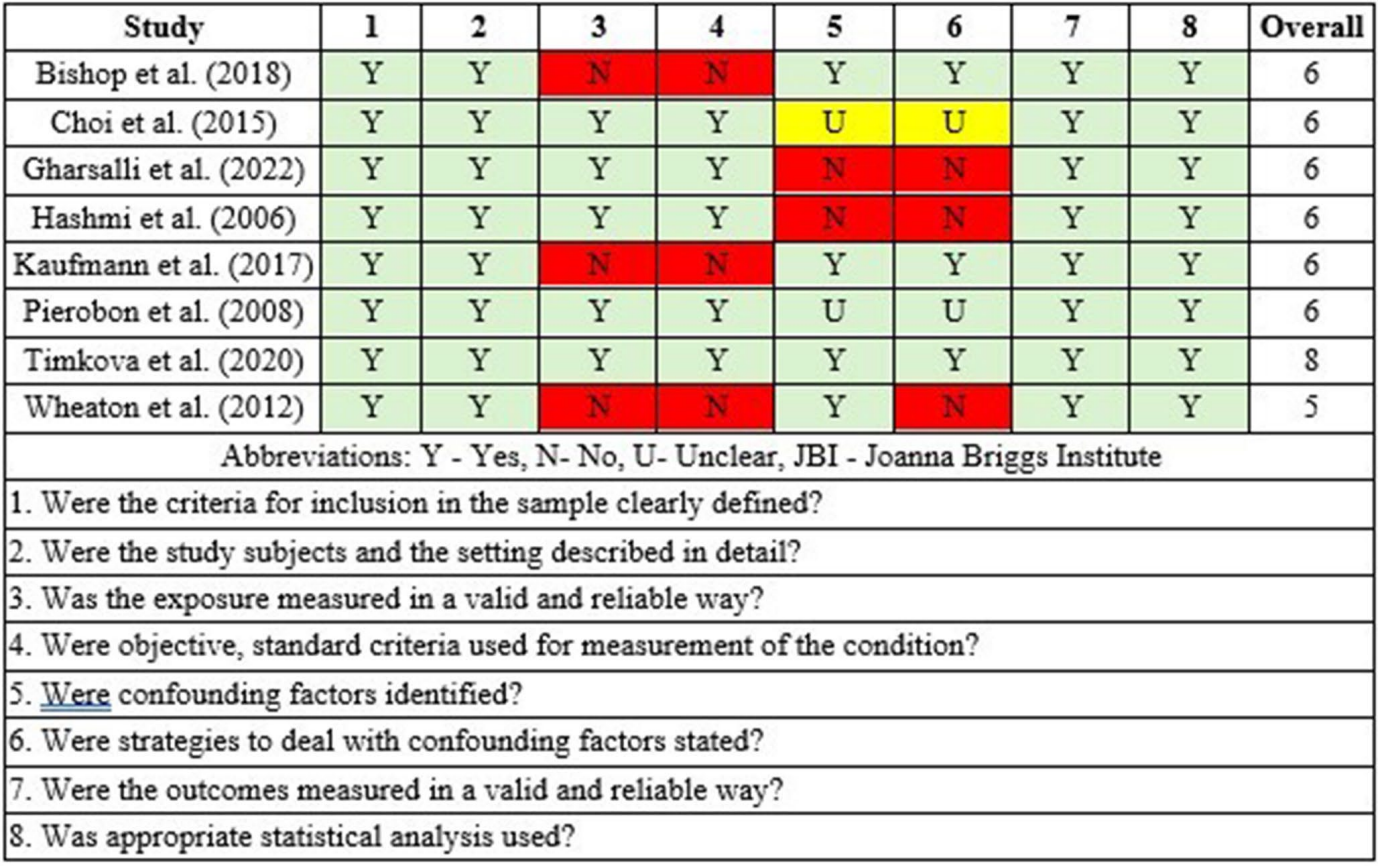
Risk of bias summary for all cross-sectional studies assessed with the JBI Critical Appraisal Tool

According to Oxford Level of Evidence criteria, the six cohort studies present level 3 evidence, while the remaining nine cross-sectional or case-control studies present level 4 evidence. Evaluation of the non-randomized studies indicated an acceptably low risk of bias (Fig. 2). The JBI assessment of the nine cross-sectional and case-control studies showed that all scored 5 or higher, suggesting good quality and a low likelihood of publication bias (Figs. 3 and S1). Additionally, a funnel plot with Egger’s test (4.6; 95% CI: −7.6 to 16.9; *p* = 0.35) showed that all six studies fell within the funnel, further suggesting minimal publication bias (Figure S2).

### Diagnosis of obstructive sleep apnea

Seven studies included patients who were diagnosed with OSA by PSG. Four studies included patients identified from national patient registries via International Classification of Diseases (ICD) codes related to sleep apnea. Two studies included participants who self-reported a diagnosis of OSA from a doctor or medical professional within the past 12 months, while a third study combined participants with a self-reported lifetime diagnosis of OSA and those reporting symptoms consistent with sleep-disordered breathing (SDB). One study included OSA patients from the Federated National Association for Respiratory Home Care in France.

### Measures of anxiety, depression, and suicidality

Five studies assessed the prevalence of anxiety; two used ICD codes, one used the Hospital Anxiety and Depression Scale (HADS), and one gathered self-reported anxiety diagnoses. Six studies assessed the prevalence of depression; two used ICD codes, two used the Diagnostic and Statistical Manual of Mental Disorders IV (DSM-IV) criteria, one used the Patient Health Questionnaire (PHQ-9), and another used the HADS. Eight studies assessed suicidal ideation; two asked respondents whether they had considered suicide in the past 12 months, and one did not specify the timeframe. The remaining five studies administered questionnaires such as the Cognitive Behavioral Assessment 2.0 (CBA 2.0) - Schedule 4, General Health Questionnaire (GHQ-28), PHQ-9, and Beck Depression Inventory (BDI-II), all of which have one or more questions related to suicidal ideation. Six studies determined the prevalence of death by suicide using ICD codes, medical records, or death certificates. One study collected data on self-reported suicide attempts over the past 12 months, and another study determined history of suicide attempts using ICD codes.

### Prevalence of anxiety, depression, and suicidality

**Fig. 4:**
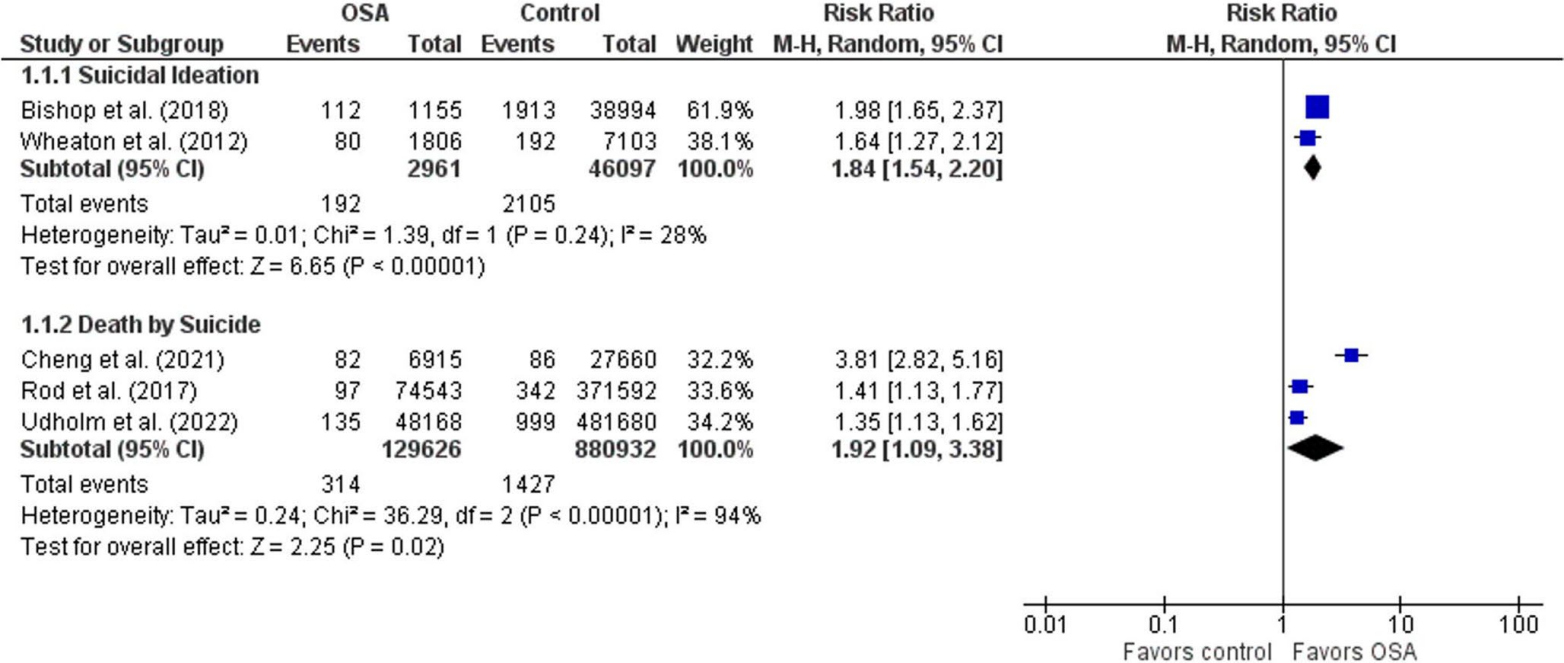
Forest plot of risk ratio for suicidal ideation and death by suicide among patients with OSA compared to the control population. The blue rectangles are study level estimates of risk ratios with 95% confidence intervals represented by lines extending from the rectangles, while the black diamonds are the pooled risk ratio based on a random effects model

Patients with OSA experienced a significantly higher prevalence of anxiety and depression than the control group (*p* < 0.001). The prevalence of anxiety was 15.9% (95% CI: 2.2–38.5%) in patients with OSA versus 5.9% (95% CI: 0.1–20.4%) in controls, and the prevalence of depression was 12.6% (95% CI: 5.0–23.0%) versus 5.3% (95% CI: 1.7–10.8%), respectively. Among eight studies, the proportion of patients with OSA (*n* = 9,060) reporting suicidal ideation was 12.4% (95% CI: 8.9%−16.3%). This was significantly different than the control population (*n* = 46,097), which had a 3.7% (95% CI: 1.9%−6.2%) prevalence of suicidal ideation (*p* < 0.001). Among two studies that assessed suicide attempts, the proportion of patients with OSA (*n* = 8,250) who attempted suicide was 1.5% (95% CI: 0.9%−2.2%), which was significantly different (*p* < 0.001) than the control population (*n* = 67,374), with a 0.9% (95% CI: 0.1%−2.2%) prevalence of suicide attempts. There was also a significant difference (*p* < 0.001) between the groups for death by suicide, which was observed in 0.3% (95% CI: 0.2%−0.4%) of patients with OSA (*n* = 207,804) compared to 0.2% (95% CI: 0.1%−0.3%) of the control population (*n* = 880,932). Among the studies that included control groups for comparison, patients with OSA had a significantly higher risk of suicidal ideation (RR = 1.8, 95% CI: 1.5–2.1) and death by suicide (RR = 1.9, 95% CI: 1.1–3.4) than the control group (*p* < 0.001). However, the risk of suicide attempts (RR = 1.7, 95% CI: 0.3–10.2) among patients with OSA was not statistically significant. Figure 4 shows a forest plot of the findings for the risk ratio of suicidal ideation and death by suicide.

To explore the impact of OSA diagnostic method on suicidal ideation, we conducted a subgroup analysis. Among studies that used clinical diagnostic criteria (e.g., PSG or ICD codes), the prevalence of suicidal ideation was 17.4% (95% CI: 10.0–26.4%) based on a sample of 601 participants. In contrast, studies relying on self-reported OSA diagnoses (*n* = 8,459) yielded a suicidal ideation prevalence of 7.3% (95% CI: 4.7–10.5%). This difference may reflect both the larger sample size and potentially broader inclusion criteria in the self-reported group. Importantly, only one study with self-reported OSA participants (*n* = 1,155), assessed suicide attempts; the remainder focused exclusively on suicidal ideation. Therefore, data on suicide attempts and death by suicide are likely to be far more reflective of individuals with clinically diagnosed OSA, who represented 215,500 of the 223,959 patients with OSA or SDB symptoms in our total sample.

## Discussion

The aim of this study was to identify relations between OSA and suicidality and its prevalence. People with OSA experienced a significantly higher prevalence (*p* < 0.001) of anxiety (15.9% vs. 5.9%), depression (12.6% vs. 5.3%), suicidal ideation (12.4% vs. 3.7%), suicide attempts (1.5% vs. 0.9%), and death by suicide (0.3% vs. 0.2%) compared to the control population. In comparison, the prevalence of suicidal ideation and suicide attempts in the United States in 2022 was 5.2% and 0.6%, respectively [30]. However, the prevalence of suicidal ideation among patients with OSA is lower than that observed in other chronic conditions, such as Parkinson’s disease and fibromyalgia with rates of 22.2% and 29.6%, respectively [31, 32].

Sleep disturbances, in general, have been associated with an increased risk of mental illness and suicide. A retrospective cohort study of 1,160 U.S. Army service members found that those with pronounced insomnia symptoms had a 3.5-fold higher likelihood of reporting suicidal ideation and were significantly more likely to have depression (12.8% vs. 1.2%) and anxiety (11.6% vs. 1.0%) than controls (*p* < 0.001) [33]. Theories to explain this association include the hypothesis that dysfunctions in biological sleep-wake systems such as the circadian system may lead to disrupted mood regulation and increased suicidality. Another hypothesis is that cognitive deficits resulting from sleep deprivation could contribute to increased risk-taking and impulsivity, which are known risk factors for suicidal behaviors [34]. The sleep disturbance that arises from OSA could impact mood and cognition in a similar manner, thus increasing the risk of suicidality. Other theories posit that OSA-related sleep architecture disruption and intermittent hypoxemia are linked to increased TNF-α and IL-6 that impair various neuropsychological and affective domains [35–37]. Hypoxemia has been linked to alterations in white matter integrity and reductions in gray matter volume. Breakdown of the blood–brain barrier (BBB) coupled with neuroinflammatory processes may compromise neuronal connectivity and accelerate cerebral small vessel pathology [36]. However, the neuropsychological and affective changes have been shown to be reversible with adequate treatment of OSA [38].

A retrospective cohort analysis based on the Taiwan National Health Insurance Research Database identified 3,025 patients diagnosed with OSA from 2000 to 2013 and found that the adjusted hazard ratio for death by suicide was 6.5 (95% CI: 5.5–7.1) compared to the control population (*p* < 0.001) [39]. In addition, a cohort study of Chinese students found that among those who reported sleep-disordered breathing, the crude odds ratio (OR) for suicidal ideation was 1.8 (1.5–2.1) (*p* < 0.001), while the adjusted OR was 1.1 (0.9–1.4). A similar trend was observed for suicide attempts, with a crude OR of 1.6 (1.2–2.2) compared to an adjusted OR of 1.0 (0.7–1.4) [40]. Moreover, in a retrospective cohort study using the National Inpatient Sample dataset of adults admitted for major depressive disorder (MDD) from 2006 to 2017, there was a significantly higher prevalence of recurrent depression (77% vs. 69%), moderate to severe depression (72% vs. 68%) and suicidal ideation/attempts (49.5% vs. 41.8%) in patients with MDD who also had OSA compared to the MDD only group (*p* < 0.001 for all) [41].

Six studies assessed the effect of continuous positive airway pressure (CPAP) treatment on suicidality, two of which were included in our meta-analysis. Chu et al. (2023) reported a lower baseline prevalence of suicide attempts among CPAP users (1.5%) than non-users (2.2%), though the difference was not statistically significant, potentially due to short treatment duration and suboptimal compliance [18]. Udholm et al. (2022) similarly found lower rates of suicide (1.4% vs. 2.1%) and self-harm (1.2% vs. 2.4%) among CPAP users, though adherence and treatment duration were not reported [27]. Wickwire et al. (2024) using device-recorded data, classified adherence based on U.S. Centers for Medicare and Medicaid Services (CMS) criteria and found that patients with sustained CPAP adherence over two years had fewer self-harm events (4.0%) than non-adherent patients (5.0%) [42]. In two cohort studies that administered either the Hamilton Depression Rating Scale (HDRS) or PHQ-9, there was no significant difference in the mean suicidality score before and after receiving CPAP [43, 44]. In a prospective cohort study of 228 OSA patients, the mean score on item 9, which assesses passive suicidal ideation, decreased from 0.28 (SD = 0.58) to 0 (*p* < 0.001) after 3 months of treatment [45]. These findings suggest adherence and treatment duration may moderate CPAP’s effect on suicidality. On the other hand, sleep surgery significantly reduces depression scores, and this correlates with sleepiness rather than the Apnea-Hypopnea Index (AHI) [46]. We have shown that both CPAP and surgery for OSA can reduce depression scores with surgery showing a higher trend. 

On assessing suicidality in OSA by gender, Chu et al. (2023) reported that the prevalence of suicide attempts among the OSA population was 1.7% overall, 1.6% among males, and 2.0% among females [18]. Hoier et al. (2022) reported that prevalence of death by suicide in the OSA population was 0.21% in males and 0.03% in females [21]. In Rod et al. (2017), the prevalence of death by suicide was also higher in males with OSA compared to females (0.15% vs. 0.09%) [25]. In two studies that assessed suicidal ideation, the prevalence was higher in females than males: 25.0% vs. 17.8% and 5.1% vs. 4.0%, respectively [26, 29]. Although the rate of suicide attempts and suicidal ideation was higher among females with OSA, the rate of death by suicide was higher among males. These trends are similar to the overall data comparing women to men in terms of suicidal ideation (4.5% vs. 4.1%) and suicide attempts (0.6% vs. 0.5%) in the United States [47]. In addition, the suicide rate among males in 2022 was nearly four times higher than that of women, with an age-adjusted rate of 23.0 per 100,000 people compared to 5.9 per 100,000, respectively [48].

It is imperative for OSA-managing providers (otolaryngologists, sleep physicians, dentists, and surgeons) to be aware of the clinical implications of these findings. A standardized psychological questionnaire is an important screening tool to detect suicidality in the clinical setting, since many patients may not otherwise disclose their distress. However, to triage the urgency of the situation and determine the resources that will be most helpful, a psychiatric interview is necessary. Upon identifying patients experiencing suicidality, it is essential to connect them with mental health professionals who can address not only their suicidality but any comorbid mental health conditions.

### Limitations and future work

Several limitations should be acknowledged. There was substantial heterogeneity across studies in terms of OSA populations, comorbidities, suicidality assessment, and control groups. Most data originated from the United States, which has a higher-than-average suicide mortality rate, potentially limiting generalizability [49]. Several studies had predominantly male samples, introducing sampling bias.

Psychiatric comorbidities such as depression and anxiety were inconsistently reported. Only a subset of studies systematically assessed these conditions, and those that did often used different tools and diagnostic criteria. Most did not adjust for mental health status, introducing potential confounding. Moreover, no studies stratified suicidality outcomes by presence of mood disorders, precluding subgroup or meta-regression analyses. We recommend that future studies consistently assess and report mood disorder comorbidities to allow for more nuanced analysis.

Suicidality was measured using a range of approaches, including validated psychiatric scales (e.g., BDI-II, PHQ-9), administrative codes (ICD-9/10), medical records, and self-report questionnaires. This variability in timeframes and measurement methods likely contributed to heterogeneity in prevalence estimates. Subgroup analysis by assessment method was not feasible due to a limited number of studies using the same tools, overlapping measurement strategies, and inconsistent reporting practices across studies. Furthermore, self-reported data may underestimate prevalence due to underreporting [50].

OSA diagnosis methods also varied. Twelve studies used clinical tools such as polysomnography or ICD codes, while three relied on self-report. Our subgroup analysis showed a higher prevalence of suicidal ideation in clinically diagnosed patients (17.4%) compared to those with self-reported OSA (7.3%). However, clinically diagnosed cases represented only 601 individuals with suicidal ideation data, while self-reported cases represented 8,459. Notably, only one self-reported study (1,155 participants) examined suicide attempts; all other self-reported studies focused on suicidal ideation. Consequently, data on suicide attempts and death by suicide were almost entirely drawn from studies using clinical diagnoses, which accounted for 215,500 of the 223,959 total OSA or SDB cases. This distinction likely influences the overall pattern of findings and should be considered in interpretation.

Sex-stratified data were also limited. While five studies provided sex-specific suicidality data (two for suicidal ideation, two for suicide death, and one for suicide attempts), this was insufficient for meta-analysis. We have narratively synthesized these findings, which underscore the need for consistent sex-stratified reporting in future studies, given established sex differences in both OSA presentation and suicidality in the general population.

Other demographic variables like age and socioeconomic status could not be analyzed due to lack of subgroup data. Finally, the cross-sectional nature of most studies prevents causal inference. While subgroup analysis helped clarify some heterogeneity, residual confounding is likely. Longitudinal research with standardized tools is needed.

There is a need for further research on suicidality and depression in patients with OSA using standardized, validated screening tools. In addition, the fluctuating nature and spectrum of suicidality cannot be captured with a single question; rather, it is important to assess suicidality at various time points and with follow-up questions. Moreover, gathering more data on potential risk factors including but not limited to socioeconomic status, mental health comorbidities, OSA severity and phenotypes that impact suicidality can improve clinical management in these patients.

## Conclusion

Patients with OSA are at risk for impaired mental health and suicidality. Our analysis shows that nearly 12% of these patients will experience suicidal ideation, and about 2% will attempt suicide. Otolaryngologists and sleep providers should have a low threshold to screen for suicidality, given the prevalence of anxiety, depression, and suicidal ideation in this population. In addition to generating more data to guide future clinical management, a standardized screening tool for suicidality in patients with OSA could improve their overall wellbeing. Evaluating impact of newer treatment options like upper airway stimulation and skeletal surgery on suicide should be considered.

## Supplementary Information

Below is the link to the electronic supplementary material.

Supplementary file 1 (DOCX 76.0 KB)

## Data Availability

The datasets generated during the current study are available from the corresponding author upon reasonable request.
