# Supplementary material for: Suicidality in adults with obstructive sleep apnea: A systematic review and Meta-Analysis
Source: Sleep Breath. 2025 Sep 29;29(5):301. doi: 10.1007/s11325-025-03462-5 (PMC12479593; doi:10.1007/s11325-025-03462-5)
Supplement: Supplementary file 1 — (DOCX 76.0 KB) [file 11325_2025_3462_MOESM1_ESM.docx]

**PubMed search strategy:**

(("obstructive sleep apnea"[All Fields] OR "sleep apnea" [All Fields] OR "apneic"[All Fields] OR "sleep-disordered breathing"[All Fields])) AND ((("suicid*"[All Fields] OR "suicidal ideation"[MeSH Terms])) OR (("suicidal"[All Fields] AND "ideation"[All Fields])) OR ("suicidal ideation"[All Fields]) OR ("suicidality"[All Fields]) OR ("suicidal"[All Fields]) OR ("suicidally"[All Fields]) OR ("suicidals"[All Fields]) OR ("suicide"[MeSH Terms]) OR ("suicide"[All Fields]) OR ("suicides"[All Fields]) OR ("suicides"[All Fields]) OR ("suicided"[All Fields]) OR ("suiciders"[All Fields]) OR ("suicidal ideation"[MeSH Terms]) OR (("suicidal"[All Fields] AND "ideation"[All Fields])) OR "suicidal ideation"[All Fields] OR ("suicidal"[All Fields] AND "behav*"[All Fields]) OR ("suicidal"[All Fields] AND "thoughts"[All Fields]) OR ("risk factors"[All Fields]) OR (("attempted"[All Fields] AND "suicide"[All Fields])) OR ("health-related quality of life"[All Fields] OR ("mental health"[All Fields]) OR ("anxiety"[All Fields]) OR ("depression"[All Fields]) OR ("self-harm"[All Fields]) OR ("mental illness"[All Fields]) OR ("distress"[All Fields]) OR ("veteran"[All Fields]) OR ("stress"[All Fields]))) Filters: Full text, Humans, Adult: 19+ years

6,052 results on 24 June 2024

**Supplement A.** Complete search strategy for PubMed database.

| **Study** | **1** | **2** | **3** | **4** | **5** | **6** | **7** | **8** | **9** | **10** | **Overall** |
| --- | --- | --- | --- | --- | --- | --- | --- | --- | --- | --- | --- |
| Veale et al. (2018) | Y | Y | Y | Y | Y | U | U | Y | Y | Y | 8 |
| Abbreviations: Y - Yes, N- No, U- Unclear, JBI - Joanna Briggs Institute | | | | | | | | | | | |
| 1. Were the groups comparable other than the presence of disease in cases or the absence of disease in controls? | | | | | | | | | | | |
| 2. Were cases and controls matched appropriately? | | | | | | | | | | | |
| 3. Were the same criteria used for the identification of cases and controls? | | | | | | | | | | | |
| 4. Was exposure measured in a standard, valid and reliable way? | | | | | | | | | | | |
| 5. Was exposure measured in the same way for cases and controls? | | | | | | | | | | | |
| 6. Were confounding factors identified? | | | | | | | | | | | |
| 7. Were strategies to deal with confounding factors stated? | | | | | | | | | | | |
| 8. Were outcomes assessed in a standard, valid and reliable way for cases and controls? | | | | | | | | | | | |
| 9. Was the exposure period of interest long enough to be meaningful? | | | | | | | | | | | |
| 10. Was appropriate statistical analysis used? | | | | | | | | | | | |

**Fig. S1** Risk of bias summary for the case-control study assessed with JBI Critical Appraisal Tool


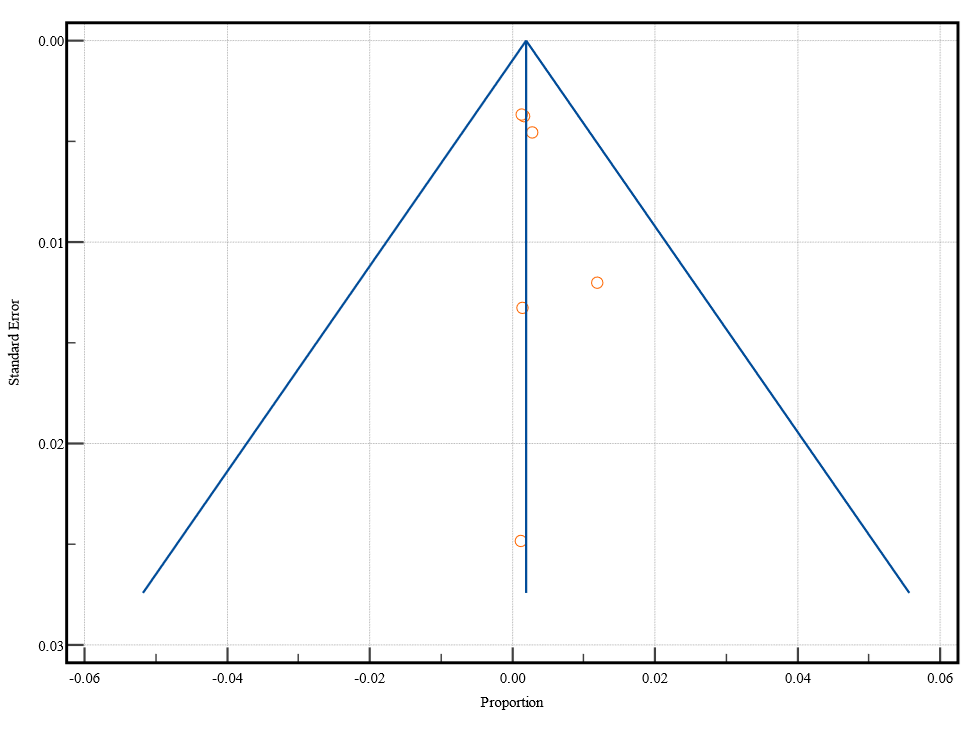


**Fig. S2** Funnel plot of studies included for analysis of overall prevalence of completed suicide. Each point represents an individual study, plotted according to the proportion of patients with OSA who committed suicide and standard error
